# Quantification of HTLV-I proviral load in experimentally infected rabbits

**DOI:** 10.1186/1742-4690-2-34

**Published:** 2005-05-23

**Authors:** Tong-Mao Zhao, Bishop Hague, David L Caudell, R Mark Simpson, Thomas J Kindt

**Affiliations:** 1 Molecular and Cellular Immunogenetics Section, National Institute of Allergy and Infectious Diseases, National Institutes of Health, Bldg #50, Room 5515, 50 South Drive, Bethesda, MD 20892, USA; 2 Molecular Pathology Unit, Center for Cancer Research, National Cancer Institute, National Institutes of Health, Bldg #37, Room 2002, 37 Convent Drive, Bethesda, MD 20892, USA

## Abstract

**Background:**

Levels of proviral load in HTLV-1 infected patients correlate with clinical outcome and are reasonably prognostic. Adaptation of proviral load measurement techniques is examined here for use in an experimental rabbit model of HTLV-1 infection. Initial efforts sought to correlate proviral load with route and dose of inoculation and with clinical outcome in this model. These methods contribute to our continuing goal of using the model to test treatments that alleviate virus infection.

**Results:**

A real-time PCR assay was used to measure proviral load in blood and tissue samples from a series of rabbits infected using HTLV-1 inocula prepared as either cell-free virus particles, infected cells or blood, or by naked DNA injection. Proviral loads from asymptomatically infected rabbits showed levels corresponding to those reported for human patients with clinically silent HTLV-1 infections. Proviral load was comparably increased in 50% of experimentally infected rabbits that developed either spontaneous benign or malignant tumors while infected. Similarly elevated provirus was found in organs of rabbits with experimentally induced acute leukemia/lymphoma-like disease. Levels of provirus in organs taken at necropsy varied widely suggesting that reservoirs of infections exist in non-lymphoid organs not traditionally thought to be targets for HTLV-1.

**Conclusion:**

Proviral load measurement is a valuable enhancement to the rabbit model for HTLV-1 infection providing a metric to monitor clinical status of the infected animals as well as a means for the testing of treatment to combat infection. In some cases proviral load in blood did not reflect organ proviral levels, revealing a limitation of this method for monitoring health status of HTLV-1 infected individuals.

## Background

HTLV-I was the first human retrovirus discovered and was isolated from cell lines derived from patients with cutaneous T cell lymphoma or adult T cell leukemia (ATL) [1,2]. Later it was found that a variety of human diseases are causally associated with HTLV-I infection, including tropical spastic paraparesis (TSP) and myelopathy/tropical spastic paraparesis (HAM/TSP) [3,4].

Previous studies of infected human subjects suggest that high proviral load is associated with increased tendency to develop HTLV-I-associated HAM/TSP, while ATL is associated with extremely high levels of provirus [5-8]. High proviral load was also found in HTLV-I infected patients with seborrheic dermatitis and severe anemia [9] and patients with rheumatoid arthritis or connective tissue disease [10]. The role of HTLV-I proviral load in the development of diseases was studied in asymptomatic carriers [11], and blood donors [12,13]. Proviral load measurement was also used to evaluate the risk of mother-to-child transmission of HTLV-I by breast-feeding [14], study the mortality in HIV-2 and HTLV-I coinfected subjects [15], monitor disease activity in HAM/TSP patients [16], count HTLV-I infected cells in healthy carriers and ATL patients [17], monitor patients following administration of interferon-α [18,19] or green tea extract powder [20], determine the genetic susceptibility to HTLV-I associated diseases [21-23] as well as determine the influence of cytokines [24,25].

Rabbit experimental infection has proven to be an excellent model of human HTLV-I infection [26-31]. Research findings made in rabbits have shed light on transmission modes, and outcomes in the infected rabbits reflect the global diversity of clinical manifestations that occur in HTLV-1 associated diseases, including a variety of cancers, immunologic diseases, and neurologic disorders [3,4]. As is the case for human beings, the majority of HTLV-1 infections in rabbits are chronic asymptomatic infections [28,29]. Data relating proviral load and disease status for the rabbit infection model would greatly enhance the utility of this experimental system and would allow further comparison to human infection. In addition the flexibility afforded by the rabbit model can allow examination of modes possible for transmission of HTLV-I infection.

In this paper we report adaptation of techniques [32] to measure HTLV-I proviral load in PBMC and organs of experimentally infected rabbits. Comparisons were made among rabbits that were inoculated either with cell-free virus, whole blood from HTLV-I infected rabbits, or with an HTLV-I cloned naked DNA. A cohort of infected rabbits monitored for as long as 2.5 yrs produced several examples of rabbits with proviral levels exceeding those established for asymptomatically infected rabbits; examination of these revealed clinical abnormalities including nephroblastoma and uterine tumors.

## Results

### Cell-free HTLV-I mediates in vivo infectivity in rabbit models

**Figure 1:**
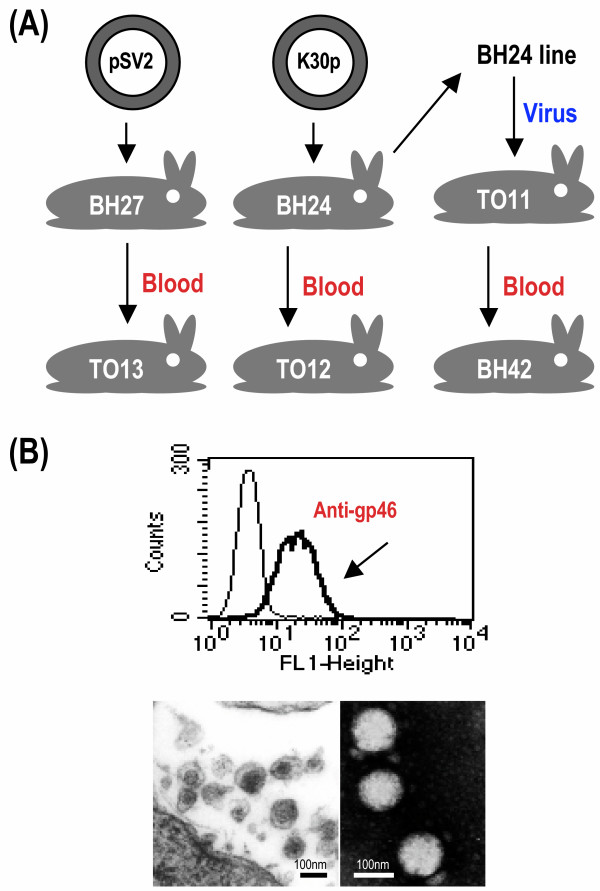
**Source and characterization of HTLV-I virions used in this study**. (A) Schematic representation of source and route of HTLV-I exposure. Rabbit BH24 was inoculated with HTLV-I clone K30p naked DNA and an HTLV-I producing cell line BH24 was derived from rabbit BH24 PBMC. Rabbit BH27 was inoculated with plasmid vector pSV2 DNA as negative control. Rabbit TO11 was infected with cell free virus prepared from BH24 cell line supernatant. Rabbits TO13, TO12 and BH42 received whole blood from rabbits BH27, BH24 and TO11, respectively. (B) Analysis of virus particles produced by cell line BH24. Fluorescence-activated cell analysis of cell line BH24 was carried out using antibodies directed against HTLV-I gp46 protein. Goat anti-mouse Ig labeled with fluorescein isothiocyanate was used as the second reagent. The figure (below) shows electro micrographs of particles isolated from supernatant of BH24. The scale bars represent approximately 100 nm. The virions concentration determined by electro micrographs was 2 × 10^10 ^per ml of BH24 cell culture supernatant. The density of particles was 1.16 g / ml measuring by ultracentrifugation on a 20% to 60% sucrose gradient. (Data not shown)

**Figure 2:**
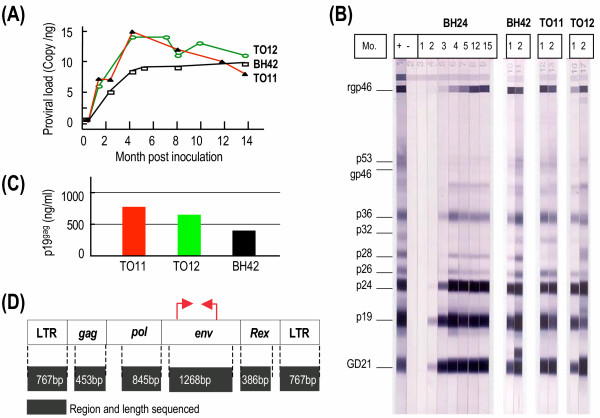
**Cell-free HTLV-I particles mediate in vivo infectivity**. (A) HTLV-I provirus was detected in rabbit PBMC; (B) HTLV-I antibodies in rabbit sera were detected using a western blot assay (Genelabs Techologies, Singapore). A goat anti-rabbit IgG conjugated with alkaline phosphatase (Santa Cruz Biotechnology, Santa Cruz, CA) was used for rabbit samples instead of goat anti-human IgG conjugate provided by the kit. Mo., month post inoculation; +, positive control serum; -, negative control serum; rgp46, HTLV-I envelope recombinant protein; gp46, HTLV-I env protein; p19 and p24, HTLV-I gag proteins; GD21 specific HTLV-I and HTLV-II epitope recombinant envelop protein. (C) HTLV-I gag p19 protein was detected in the culture supernatants of rabbit PBMC taken at one month post inoculation. (D) Schematic representation showing the regions sequenced. The target regions were amplified by PCR and purified PCR products served as templates for direct sequencing. Stable transmissions of HTLV-I sequence fragments in rabbit BH24, TO11, TO12 and BH42 were observed. No mutation was detected in the analyzed LTR, *gag*, *pol*, *env*, and *rex *regions for the period of observation up to 20 months. The red arrows indicate the primers used to amplify an env fragment in real-time QPCR assay.

An HTLV-I producing cell line BH24 was derived from rabbit BH24 inoculated with HTLV-I molecular clone K30p naked DNA. HTLV-I env protein gp46 was detected on the surface of BH24 cells and HTLV-I virions isolated from BH24 cell line have normal size and density (Figure 1). Cell free HTLV-I prepared from BH24 cell culture was injected intravenously into rabbit TO11 and rabbit TO12 was given whole blood from BH24. After infection was established rabbit BH42 received blood from TO11 (Figure 1). After two weeks post inoculation all three rabbits including TO11, TO12 and BH42 produced HTLV-I antibodies, HTLV-I provirus was detected in their PBMC, and HTLV-I gag p19 protein was detected in PBMC culture supernatants (Figure 2). These data indicated that cell-free HTLV-I can mediate infectivity in rabbits as does infected blood. In order to determine whether the HTLV-I mutated during the course of infection and transfer, provirus from the rabbits (BH24, TO11, TO12, and BH42) was subjected to sequence analysis at three time points: 8, 12 and 20 months post inoculation. Based on previously observed sequence differences in HTLV-1 regions selected from LTR, *gag*, *pol*, *env *and *rex *genes were analyzed. For each isolate 4,486 bases were compared and no differences from the original K30 clone were detected for the period of observation. These data gave confidence that proviral load studies may be conducted with little concern for effects of mutations on primer recognition of the provirus.

### Proviral load in PBMC of HTLV-I infected asymptomatic rabbits

The proviral load was determined using a real time PCR-based QPCR assay, in which HTLV-I *env *gene was selected as an amplification target. To determine the sensitivity for this assay, scalar dilutions of K30p clone DNA ranging from 1 to one billion (10^9^) copies were analyzed. The results indicate that a positive signal was consistently detected at HTLV-I DNA concentrations above 1 copy per ng of DNA. Based on these results the limitation of this assay was considered to be 1 copy of HTLV-1 proviral DNA per ng of genomic DNA.

**Figure 3:**
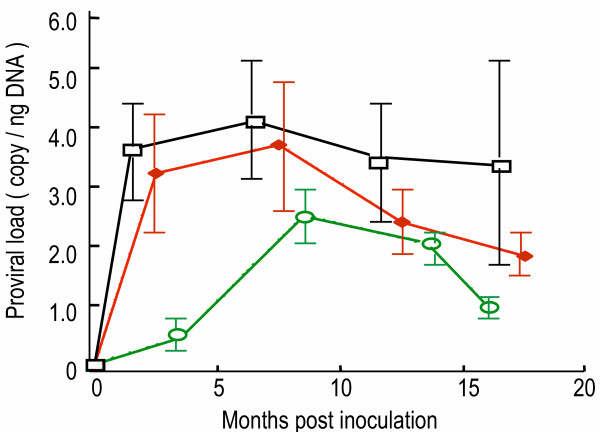
**HTLV-I proviral load in PBMCs of asymptomatic rabbits infected with HTLV-I by different routes**. Rabbits inoculated with: (1) whole blood (open squares, n = 29), (2) cell free virus (solid diamonds, n = 19), and (3) naked K30p DNA (open circles, n = 5). Proviral load is present as mean and standard errors (error bars). Probability for statistically significant: (1) vs. (2), P = 0.05; (1) vs. (3), P < 0.001; (2) vs. (3), P < 0.01.

Fifty-seven rabbits infected by different routes and using different sources of HTLV-I were monitored for proviral load over a period of 75 weeks at two to four weekly intervals (Figure 3). The highest average proviral load was observed in PBMC from rabbits inoculated with HTLV-I infected whole blood and values peaked at 30 weeks post inoculation. Rabbits injected with HTLV-I naked DNA produced lower levels of provirus and did not reach maximum levels until later than rabbits in the other groups. Provirus loads were intermediate in rabbits injected with cell-free virus and reached maximum levels early as did those given infectious blood. The proviral load peaked around 30 weeks post infection in blood of all rabbits and decreased after that time.

Average proviral load measured for HTLV-I infected asymptomatic rabbits was compared with reported data for human samples (Table 1). When all data are converted to the same units, that is, copies of provirus per nanogram of DNA, a close similarity in levels of provirus is seen between the experimentally infected rabbits and the asymptomatic human subjects.

**Table 1:** Comparison of proviral load in HTLV-I infected asymptomatic rabbits and human samples

Subjects	Number tested	Copies/ng DNA*	Ref.
Rabbits inoculated with			
Naked K30p DNA	5	1.5	
BH24 cell-free virus	19	2.8	
Whole blood	29	3.7	
pSV2 plasmid DNA	1	0	
			
Human Asymptomatic carriers	200	1.8	[5]
	15	1.0^†^	[36]
	83	2.7^†^	[20]
	120	2.4	[12]
			
Human HAM/TSP	202	12.0	[5]
	15	8.8^†^	[36]
	9	19.9	[19]
			
Human ATLL	4	47.4^†^	[37]

* The mean or median (^†^) of proviral loads are present as HTLV-I copy number / ng of genomic DNA prepared from PBMC. The rabbits samples were collected after 2 months post inoculation. All published data were converted to this format on the basis of one ng of genomic DNA corresponding to approximately 150 cells.

**Figure 4:**
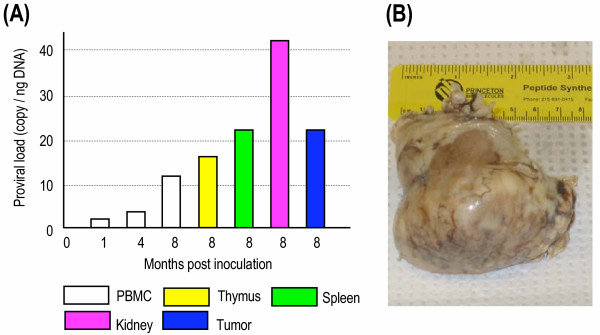
**Proviral load in rabbit TO9 that developed a renal nephroblastoma**. Rabbit TO9 was inoculated with cell-free HTLV-I prepared from HTLV-I-producing rabbit cell line RH/K30. The rabbit was necropsied at 8 months post inoculation due to renomegaly. (A) HTLV-I proviral load was determined in PBMC, selected organs, and in both neoplastic (tumor) and non-neoplastic (kidney) regions of the kidney (B) Rabbit kidney, gross photograph of nephroblastoma.

In the course of collecting proviral load data one rabbit (TO9) showed an unexpected increase from about 3 copies/ng in a sample taken at 4 months to more than 10 copies/ng at 8 months post infection with cell-free virus. Examination of the rabbit revealed an enlarged kidney, which upon necropsy, was a nephroblastoma. Tissues selected from rabbit TO9 and tested for provirus revealed elevated levels in the thymus, spleen and the tumor dissected from the kidney (Figure 4). Proviral load in the non neoplastic portion of the kidney was 4 times higher than the rabbit's own blood lymphocytes and 10 times the average blood value for all the rabbits.

### HTLV-I provirus load in rabbit organs and tumor during early and chronic phase of infection

In order to determine proviral loads in major organs from animals infected by different protocols, samples taken at necropsy were analyzed. Table 2 shows the distributions of HTLV-I provirus in rabbit organs. In general the levels in the organs tested were lower than those of the PBMC taken at the same time. However, there are sporadic instances of high proviral load in certain samples, (for example, the thymus, skin and heart samples from BH19 and the spinal cord of T4-9) but no consistent pattern emerged from this analysis. Rabbit BH76 exhibiting a typical PBMC proviral load had an increased level of provirus in its uterus (Table 2). The proviral load within the benign uterine endometrial tumor collected at necropsy was greater than adjacent nontumorous uterine endometrium, by contrast. A somewhat similar relationship among proviral loads in blood, tumor and nonneoplastic adjacent endometrium was observed in rabbit BH25 with a uterine adenocarcinoma (Table 2).

**Table 2:** Distribution of proviral load in PBMC and organs of HTLV-I infected rabbits*

	Inoculated with						
	
	Naked DNA			Cell-free virus		Whole blood	
			
ID (Mo.)^†^	BH19(30)	BH21(30)	BH25(30)	T4-9(11)	BH69(19)	BH76(17)	BH89 (18)
PBMC	7.5	3.9	1.4	6.7	1.3	2.2	8.7
Thymus	22.6	0.4	0.6	0.1	0.3	0.5	1.0
Liver	1.3	0.3	0.3	0.3	0.4	1.8	0.8
Spleen	1.6	0.4	0.1	0.3	0.3	1.7	ND
Skin	23.0	0.3	0.2	4.1	0.2	2.3	0.5
Heart	12.7	2.5	0.3	12.0	0.3	3.1	1.9
Lung	1.8	0.8	0.8	ND	0.7	1.5	2.0
Kidney	3.4	0.5	1.6	0.2	0.5	0.9	1.4
Uterus	3.2	3.0	0.3	ND	1.2	5.0	0.5
Spinal cord	1.7	0.2	0.9	50.5	0.4	0.3	1.2
Brain	0.1	0.3	ND	6.9	ND	ND	ND
Tumor ^‡^	NA	NA	1.8	1.9	NA	11.0	NA

* Proviral load was reported as copy number per ng of genomic DNA. ND, not determined.

^† ^Rabbit identification number (month post inoculation).

^‡ ^Rabbits T4-9 had a uterine leiomyoma, BH76 had benign endometrial dysplasia, and BH25 developed a uterine adenocarcinoma. NA, not applicable.

Rabbit T4-9 also harbored a uterine neoplasm, however the lesion was not within the endometrium, but resided in the tunica muscularis and was classified as a uterine leiomyoma. This rabbit was found to have an elevated level of PBMC provirus, while the proviral load in the tumor was 1.9 copies per ng of DNA. Interestingly this rabbit's spinal cord showed an unusually high proviral load, which may have been what the blood value reflected.

The proviral load measurement also provided a means to track inoculated rabbits infected with HTLV-1 cell lines known to cause an ATLL-like disease. In the rabbits receiving a high dose of RH/K34 cells known to result in experimental ATLL, proviral loads observed in the lung, kidney and thymus were well above the range established for asymptomatically infected rabbits (Table 3). One of the two rabbits sampled had high values for liver and spleen while the same organs of the other were negative for provirus. The thymus, lung, and kidney of both rabbits had high levels of provirus, consistent with data obtained by histologic studies of organs from these rabbits with experimental ATLL [30].

**Table 3:** Proviral load in rabbits inoculated with RH/34 cells*

	Cells inoculated		
	
	2 × 10^6^	2 × 10^8^	
		
ID	BH121	BH120	BH101
PBMC	0.5	1.0	ND
Thymus	1.6	88.3	26.3
Live	2.5	207.0	0.5
Spleen	0.0	25.7	0.4
Skin	0.1	6.9	4.8
Heart	0.3	7.3	24.5
Lung	5.1	221.6	217.1
Kidney	0.7	213.0	99.6

* Proviral load was reported as copy number per ng of genomic DNA. The samples were collected at 96 hours post inoculation. ND, not determined.

## Discussion

The present study describes infection of the rabbit with HTLV-1 by several different modes and compares the results of infection. In addition the data show that virus sampled at various time post infection retained the sequence of the original HTLV-1 clone indicating that variations in response to infection cannot be attributed to virus mutation. The data here show reproducible *in vivo *infectivity of rabbits using naked DNA, cell-free virus, infected cell lines or whole blood obtained from HTLV-1 infected rabbits. As previously reported most infections were asymptomatic although certain rabbits monitored for extended periods did develop tumors. An exception to the asymptomatic infection involved rabbits challenged with high doses of the infected cell line RH/K34 [30]; these rabbits succumbed to an aggressive leukemia-like disease within several days.

The assay used to measure provirus load in human patients was adapted for use in the rabbit model and levels of HTLV-1 in blood and parenchymal organs were measured for rabbits infected using different inocula. The rabbits inoculated with either infected whole blood or with cell-free virus showed similar levels of proviral load. In these animals the provirus quickly assumed maximum values and stayed high for about 30 weeks then dropped somewhat to levels that were maintained throughout the period of observation which was up to 70 weeks. A different pattern of provirus load was seen in rabbits infected with naked HTLV-1 DNA clone. Provirus load levels rose slowly in these animals and after reaching maximum levels around 30 weeks began to decline. Average provirus load in the rabbits infected with DNA reached values approximately half those infected with blood or cell free virus.

Comparison of the provirus load values observed for the rabbits were compared to those reported for human subjects infected with HTLV-1. A close correlation with levels in asymptomatic infected humans was seen. Levels in rabbits infected using whole infected blood were slightly higher on average than the human average values but in general the levels suggest that control of infection is similar in the two species.

In several incidents the provirus level rose in an unexpected manner in infected rabbits. One of these, rabbit T09, showed an increase in blood level of provirus to over 10 copies per nanogram of DNA which is about 4 times normal value. Physical examination and subsequent radiograph of the rabbit revealed an enlarged kidney which upon necropsy was shown to harbor a large tumor. The tumor was a nephroblastoma and DNA from it had about 20 copies of provirus per ng. Examination of DNA from lymphoid tissue and the kidney tissue indicated high levels of provirus. In all cases these were considerably greater than the blood levels of provirus. The kidney levels were higher than those of the tumor.

Several other rabbits in the study developed signs that warranted examination and these animals were sacrificed and their organs examined and provirus load determined. For most organs the level of provirus was at the limit of detection and could be dismissed as negative or due to slight amount of contamination by blood. Exceptions to this were seen and point to unusual consequences of infection. For example BH19 had high blood levels and its skin was shown to harbor exceptionally high provirus load. It is tempting to speculate that this animal was enroute to developing cutaneous signs of infection as has been seen in the rabbit model [31]. Rabbit T4-9 had a provirus load of 6.7 copies per ng at sacrifice and examination revealed spinal cord and brain with high provirus load. A neurologic consequence of this infection may be predicted in patients with HAM [38,39]. Of three rabbits with uterine tumors one had high levels of provirus in the tumor tissue whereas two others did not. In the cohort of infected rabbits four developed tumors (3 uterine and 1 kidney) and of these 2 had elevated blood levels of provirus. While this number of events is too low to draw a conclusion about correlation between tumor development and proviral load it is interesting that examination of every rabbit with elevated blood provirus revealed either organ infection or development of a tumor.

In this study, we successfully used a quantitative assay to measure the proviral load of HTLV-1 in PBMC and organs from several cohorts of infected rabbits. Validation of this adapted methodology strengthens the utility of this model for the study of human patients with chronic HTLV-1 infections. Proviral load measurements were made in rabbits infected by different methods; proviral loads from this series of animals infected by different methods documented levels that appeared to stratify according to source of inoculum. Such findings suggest potential of this model for study of HTLV-1 transmission and its relationship to differences in infectious load. In addition to monitoring rabbits that were asymptomatic carriers, proviral load was determined in a subset of rabbits with ATLL-like disease. Data suggested proviral load varied according to tissue compartment, to severity of leukemic infiltration of organs, and to original inoculum dose. If substantiated in larger studies, assay for proviral loads in tissue compartments may reveal additional insight into pathogenesis of lesions in ATLL [12]. Additionally, preclinical therapeutic strategies and drug efficacy designed to combat retroviral infections can be monitored in this system with greater confidence by measuring proviral load status as a response to treatment.

## Materials and methods

### Animals

The female New Zealand White rabbits were used in this study. Six rabbits were given four 100 μg intramuscular injections of HTLV-I clone K30p naked DNA [33] at biweekly intervals [34]. Twenty-one rabbits were tested for cell-free virus infectivity by intravenous inoculation with 1 to 3 ml of virus preparation containing 1 to 5 × 10^12 ^copies of viral RNA. A total of 30 rabbits received 3.0 ml of whole blood obtained from HTLV-I infected rabbits. Three rabbits were inoculated with rabbit cell line RH/K34, which induces lethal leukemia-like disease in rabbit in high dose inoculation. Infection in rabbits was monitored by the presence of anti-HTLV-1 antibody, virus production in PBMC culture, and detection of viral sequences in PBMC and organs as previously described [34]. The health status of all rabbits on study was monitored by physical examination at time of blood drawing.

### Cell lines

The RH/K30 and RH/K34 cell lines were derived by infection of rabbit peripheral blood mononuclear cells using human HTLV-I infected cell line, MT-2. The BH24 cell line was derived from a rabbit inoculated with an infectious HTLV-I molecular clone K30p naked DNA. BH24 cell line is available for research purposes from the AIDS Research and Reference Reagent Program (McKesson BioServices, Germantown, MD)

### Preparation of cell-free HTLV-I

Cell-free viruses were prepared from the culture supernatant of HTLV-I producing cell lines. Cells and debris were removed from supernatants by centrifugation at 800 g for 10 min, and then passed through a 0.22 mm filter (Millipore Corporation, Bedford, MA). The filtrates were concentrated to15 to 20 fold through a centrifugal filter device with 100 NMWL membrane (Millipore Corporation, Bedford, MA). The virus stock preparation was stored at -80°C until use. The virus quantitation was measured by a real-time QRT-PCR assay. Thawed virus preparations lost binding activity within several hours unless kept at 4°C [35]. HTLV-I gag p19 protein was determined by a commercial ELISA test (Cellular Product Inc. Buffalo, NY).

### Preparation of genomic DNA

The PBMC genomic DNAs were isolated from EDTA-treated blood samples using Wizard Genomic DNA Purification Kit (Promega Corporation, Madison, WI). The organ DNAs were prepared using DNeasy Tissue Kit (Qiagen, Hilden, Germany).

### Quantification of HTL V-I proviral load

Two sequence-specific primers that detect HTLV-I *env *region were used to amplify a 185 bp fragment. The sequences of HTLV-I *env *primers are: 5'-ATC CAC TTG GCA CGT CCT ATA-3' (nt 5890–5910, GenBank accession no. L03561) and 5'-GCA GGA TGA GGG AGT TAT GTC-3' (nt 6054–6074). The dual-labeled fluorescent probe was FAM -5'-CTT TAC CCA TCG TTA GCG CTT CCA GCC CCC-3'-BHQ1 (nt 5954–5983). Rabbit beta-globin DNA quantitation was performed in parallel on all samples in order to determine the amount of cellular DNA present and was used as an endogenous reference to normalize variations due to differences in the PBMC count or DNA extraction. A 187 bp fragment of the rabbit beta-globin gene was amplified by forward primer 5'-GGT ATC CTT TTT ACA GCA CAA C-3' (nt 372–393, GenBank accession no.V00882) and reverse primer 5'-CAG GTC CCC AAA GGA CTC G-3' (nt 531–549) in a real-time PCR assay. The fluorogenic probe used to detect rabbit beta-globin gene was 5'Quasar 670 - CCT GGG CTG TTT TCA TTT TCT CAG G - BHO-2, 3' (nt 471–495). Both the primers and probes were synthesized by a commercial company (Biosearch Technologies, Inc., Novato, CA)

HTLV-I *env *and rabbit beta-globin gene fragments were amplified separately with an Mx3000P Real-Time PCR System (Stratagene, La Jolla, Calif.) in 50 μl reaction mixture consisting of 10 μl of DNA sample, 25 μl of Brilliant QPCR Master Mix (containing PCR buffer, SureStart Taq DNA polymerase) (Stratagene, La Jolla, Calif.), 10 pmol of each primer, and 5 pmol of TaqMan probe. Thermal cycling conditions were as follows: 95°C for 10 min, and 45 cycles of 95°C for 30 s, 55°C for 1 min, and 72°C for 30 s. Each sample was analyzed in duplicate, and HTLV-I proviral load was calculated at the copy number of each per ng of genomic DNA.

## List of abbreviations

ATLL, adult T-cell leukaemia/lymphoma

HAM/TSP, HTLV-I -associated myelopathy/ tropical spastic paraparesis

PBMC, peripheral blood mononuclear cells

QPCR, quantitative polymerase chain reaction

QRT-PCR, quantitative reverse transcription-polymerase chain reaction

## Competing interests

The author(s) declare that they have no competing interests.
